# Who owns reefer vessels? Uncovering the ecosystem of transshipment in fisheries

**DOI:** 10.1126/sciadv.adn3874

**Published:** 2024-10-11

**Authors:** Frida Bengtsson, Jean-Baptiste Jouffray, Shinnosuke Nakayama, Erik Zhivkoplias, Colette C. C. Wabnitz, Robert Blasiak, Elizabeth R. Selig, Henrik Österblom

**Affiliations:** ^1^ Stockholm Resilience Centre, Stockholm University, 106 91, Stockholm, Sweden.; ^2^ Stanford Center for Ocean Solutions, Stanford University, Stanford, CA 94305, USA.; ^3^ Institute for the Oceans and Fisheries, University of British Columbia, Vancouver, BC V6T 1Z4, Canada.; ^4^ Anthropocene Laboratory, Royal Swedish Academy of Sciences, 104 05 Stockholm, Sweden.

## Abstract

A central barrier to effective governance and accountability in fisheries is the limited transparency of corporate ownership. Transshipment—the transfer of catches, fuel, parts, or crew between fishing and cargo vessels known as reefers—is often criticized for its opacity and poor governance. Better insight into the beneficial ownership of vessels involved in transshipment and their operational patterns could lead to more effective management. Our study presents a publicly accessible database of reefers’ owners, operators, and flags. We identified 569 individual reefers and found that Russian and Chinese owners control 26 and 20% of the global reefer fleet, respectively. Results also show that 65% of all reefer vessels fly the flags of Russia, Panama, or China. This high level of consolidation suggests considerable leverage for enhancing transparency and governance. Our findings highlight the potential for reforming existing transshipment practices through collaboration among owners, flag states, fishery regulators, and scientists.

## Teaser

Identifying beneficial owners of reefer vessels provides opportunities for engagement toward improved fishery management.

## INTRODUCTION

The past two decades have seen increased scientific, regulatory, and public attention paid to the sustainability of fisheries (*1*–*4*), including efforts to eliminate illegal, unreported, and unregulated (IUU) fishing (*5*–*10*). These practices persist because of the vastness of the ocean, the challenges of enforcement, and the associated costs and benefits for vessel owners who evade or break existing rules and regulations (*11*). Increasing management efforts and cooperation at the national and international levels have been directed at fishing vessels to reduce the prevalence of IUU fishing. While the role of transshipment remains less well understood and in need of reform (*12*, *13*).

**Fig. 1.**
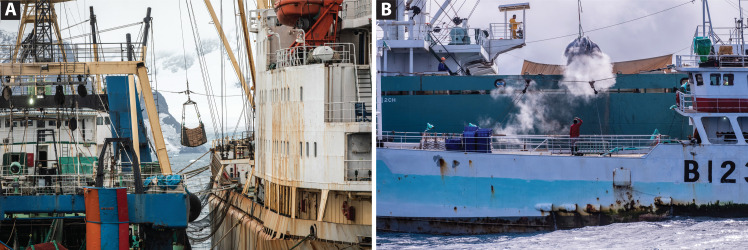
At sea transshipments. (**A**) Ship-to-ship transfer between a krill fishing vessel and Russian-flagged reefer Pamyat Kirova (IMO 8701040) operated by Baltmed Reefer Services (Greece) and owned by Laskaridis (Greece) at the South Orkney Islands, Antarctica (February 2020). (**B**) Frozen tuna is transferred from the Hung Hwa 202, a longliner from Chinese Taipei, to the Panama-flagged Hsiang Hao (IMO 9797656) operated by Toei Reefer Line (Japan) and owned by Ryoma Marine Transport Corporation (USA) in the South Atlantic Ocean (September 2019) (data file S1). Credits: (A) Andrew McConnell/Greenpeace and (B) Tommy Trenchard/Greenpeace.

Transshipment, defined by the Food and Agricultural Organization (FAO) of the United Nations (UN) as “the direct transfer of any quantity of fish onboard from one vessel to another vessel regardless of the location of the event, without the fish being recorded as landed” and encompassing the associated “transfers of supplies, crew and other materials” (*14*). Transshipment commonly involves conventional reefers or refrigerated cargo vessels, hereafter “reefers,” interacting with fishing vessels (Fig. 1).

The practice allows fishing vessels to remain at sea for extended periods by supplying food, changing crew, refueling, and transporting catches back to ports instead of fishing vessels doing so themselves. It has gained prominence alongside the spatial expansion of fisheries and technological advances related to processing, freezing, and storing fish onboard fishing vessels (*15*, *16*). Transshipment can also occur within ports, particularly in the context of transporting fish species caught using gear types that are subject to at-sea transshipment prohibitions (*17*) or in conjunction with scheduled port visits for crew rest and exchange, reprovisioning, and essential maintenance.

The practice of transshipment is common in multiple fisheries and highly seasonal and often follows the migration patterns of target species (*18*). It is governed by specific Conservation and Management Measures (CMMs) established by international agreements in Regional Fisheries Management Organizations (RFMOs) or through national regulations, including several with chain-of-custody verifications (*19*, *20*). RFMOs are a crucial component in global fishery governance, with a mandate to regulate and manage fishing for migratory and straddling fish stocks and associated activities such as transshipment (*21*). Although transshipment is a common and regulated practice, it has been repeatedly associated with illegal fishing and crimes at sea (*22*–*26*). Reefers can facilitate illegal activities by assisting fishing vessels in evading regulations, enabling illegally caught species to be mixed with legally caught fish, or obscuring the origin of seafood (*27*, *28*). By extending the time fishing vessels can spend at sea, the practice reduces the possibility of monitoring the labor conditions of crew members working onboard (*29*, *30*). But without it, fuel costs would be much higher, potentially incentivizing cuts to labor costs (*31*). A global assessment of fishery-related offenses between 2000 and 2020 identified 6853 events or “incidents,” of which 140 were classified as illegal transshipment (*32*). Even in cases of legal transshipments, the practice may obscure the origins and destinations of catches and undermine seafood traceability (*33*, *34*).

Leveraging the rapid increase in the availability of legally mandated vessel monitoring technologies (*35*) and public datasets, recent efforts to understand transshipment have primarily focused on identifying the geographical location where reefers and fishing vessels meet and the flags that they operate under (*33*, *36*–*39*). Three types of data have been vital in this context: (i) Automatic Identification System (AIS) data, which provide geolocation information for ocean-going vessels (*35*); (ii) International Maritime Organization (IMO) numbers, which are unique identifiers attached to the hull of vessels (*40*); and (iii) Maritime Mobile Service Identity (MMSI) numbers, which are nine-digit electronic identifiers associated with the vessel’s communication equipment (*41*). These datasets have enabled researchers and Non-Governmental Organizations (NGOs) to identify and analyze vessel movements, fishing behavior, and associated practices, including transshipment (*42*–*44*). Notably, Park *et al.* (*43*) identified about 2000 support vessels engaged in transshipment and refueling at sea as part of a broader assessment of fishing compliance and reflagging patterns. Similarly, Petrossian *et al.* (*33*) identified 130 carrier vessels as “central” in the networks of global transshipment activities, concentrating primarily in the Eastern Central Pacific and the northern section of the Southeast Pacific. A common aspect across these studies is the emphasis on tracking patterns across reefer activity to understand transshipment operations better. Here, we use a consistent methodology to identify reefers, explore the legal entities underpinning transshipment, and identify the ownership structure within the transshipment industry. Knowing where transshipments occur and who is involved can help inform pathways for improved accountability.

The UN Convention on the Law of the Sea grants any state the right to assign nationality to vessels (Article 91) and allow them to fly its flag, along with the responsibility to ensure safety and compliance with international regulations (Article 94). In practice, this has resulted in a diversity of ship registries and flag states exercising monitoring and control functions to varying degrees. The shipping and fishing industries, in turn, enjoy broad flexibility in deciding where to operate their vessels and under which flag (*45*–*47*). Transshipment exists at the nexus of two distinct industries: the shipping industry (reefer vessels) and the seafood industry (fishing vessels). Each is subject to its own regulatory landscape and has a unique set of owners and operators.

Transshipment requires a high degree of coordination and sophistication, ultimately relying on companies that own or operate reefers to position the right vessel in the right place at the right time (*18*, *48*). Owners of reefers play a critical role because they ultimately decide where their vessels are deployed and which flags they fly, thereby determining the jurisdiction and states responsible for regulating their operations. In this study, we aim to answer three key questions: Who owns reefer vessels involved in transshipments, where do they operate their vessels, and who do they meet with? To answer these questions, we first draw upon multiple data sources, including public and maritime intelligence databases, to compile a publicly available database of reefers active between 2017 and 2022 and identify their owners. We link these owners to the specifics of their operations at sea, including main areas of activity, flag states, gear types (e.g., trawl, gillnet, and longlines), and the flags of the fishing vessels that they encounter. We then outline how this information can enhance corporate transparency and accountability, revealing previously unidentified leverage points for engaging with corporate actors to improve transshipment governance and contribute to the sustainability of fisheries.

## RESULTS

### Reefer owners

We identified 569 individual reefers on the basis of their vessels’ IMO numbers, displaying 724 unique MMSI numbers and associated with 324 beneficial owners, hereafter “owners,” from 2017 to 2022. This apparent mismatch arises because some owners own multiple vessels, and a given reefer can have multiple MMSI numbers over its lifetime due to changes in ownership or flag (Box 1).

Box 1. From fruit to fish: ownership and flag changes in the reefer fleet.

The Yong Xiang 9 (IMO 9158537) and Shen Ju (IMO 918985) are two reefers now owned by East Maritime Services Company Limited, based in Malaysia. Both reefers were previously involved in the fruit trade and first encountered a fishing vessel after being sold to East Maritime in 2018. Yong Xiang 9, launched in 1998, was previously owned by Seatrade Holding with the name Agulhas Stream and sailed under the flag of Curaçao. The vessel was sold in 2018 to East Maritime and reflagged to Panama. The Shen Ju was previously named Polarstream and flagged to Liberia until 2018 when MPC Group sold it to East Maritime and re-flagged it to Panama. By the time the analysis for this paper concluded in November 2023, both reefers had changed flags again to Vanuatu, according to Lloyds Seasearcher (data file S1).

The single largest owner, a Greek ship-owning conglomerate controlled by members of the Laskaridis family, hereafter Laskaridis, owned 46 reefers during our study period. They were followed by China National Agricultural Development Group and its 20 reefers. Russia had the largest number of owners (119), followed by China (65), Chinese Taipei (32), Hong Kong SAR (19), Japan (14), and South Korea (14). Overall, owners in Russia (26% of all reefers) and China (20%) alone controlled nearly half the number of reefers engaged in transshipment between 2017 and 2022. They were followed by owners based in South Korea (10%), Greece (7%), Japan (7%), Chinese Taipei (6%), Hong Kong SAR (6%), Norway (4%), and The Netherlands (3%). We also identified the operators of reefer vessels, finding that 178 vessels were operated by entities different from their beneficial owners, although typically a subsidiary of the owner (data file S1). No single operator was contracted by multiple owners. However, operators could be connected through reefer pools (Box 2) (*49*).

Box 2. Reefer pools.

Reefers can be used in a profit-sharing scheme by a group of reefer owners called a reefer pool, where all vessels inside the pool share revenues (*48*, *49*). GreenSea is a Belgium-based reefer pool established as a joint venture between the Norwegian shipping company Green Shipping AS and the Dutch-based Seatrade Group. Ships operated by GreenSea collect fish at fishing grounds and transport them from port to port. They frequently operate in Mauritania, Senegal, Namibia, Chile, and the North Atlantic. The company also carries meat and poultry, fresh fruit in season, and chilled and frozen juice. They backhaul cargo such as bagged goods, chemicals, rolling stock, machinery, steel, and lumber (*18*, *62*, *74*). Frigoship Chartering, specialized in vessel chartering, oversees ships enrolled in the Alpha Reefer Transport (ART) pool. The ART pool consists of five ship owners transporting meat and fish. The focus is fish from West Africa, tuna from the Seychelles, and squid from the Falkland Islands. They also offer time charters for the Pacific tuna market. The reefer pool is engaged in both transport between ports and from fishing grounds (*18*, *75*, *76*).

**Fig. 2.**
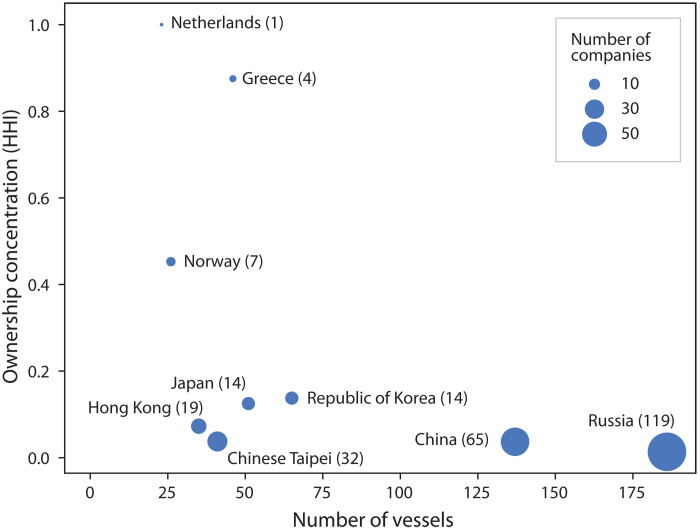
Owner concentration in the reefer industry. The degree of market concentration in each reefer owner’s state is measured with the Hirschman-Herfindahl index using the number of reefers as a proxy unit. Higher values indicate market dominance by a few companies, and lower values suggest competitive markets. The dot size represents the number of owners in each country (also indicated in parenthesis). Owner states with fewer than 10 vessels are excluded (data file S1).

The relative concentration of ownership varied considerably across the above-listed states. For instance, reefer owners are highly concentrated in The Netherlands [Hirschman-Herfindahl index (HHI) = 1] and Greece (HHI = 0.87), with a few owners holding a substantial percentage of each country’s fleet. In contrast, reefer owners in Russia (HHI = 0.013) and China (HHI = 0.036) held a more or less equal share and the reefer market displayed patterns consistent with being more competitive (Fig. 2). The single largest reefer-owning company in Russia controlled about 5% of the country’s reefers, while the largest in China controlled 10%.

Between 2017 and 2022, the 10 most active owners, based on the number of encounters (as an indicator of their role as transshippers within the industry), accounted for 23% of all reefer activity globally. The Laskaridis consistently ranked as either the most active or second most active owner in any given year based on the number of encounters, despite Greek-owned vessels only making up 7% of the total reefer fleet. They were followed by owners based in Russia and China (fig. S1).

### Flags, operations, and fishing vessel interactions

#### Flags used by reefer owners

Only three flag states—Russia (196 reefers), Panama (189), and China (82)—accounted for 65% of flags used by reefers between 2017 and 2022. These flag states were followed by South Korea (37 reefers), Liberia (35), and Vanuatu (28). Around 90% of reefers owned by companies in Russia used the Russian flag, while only 50% of owners in China used the Chinese flag, followed by the flag of Panama (32%). None of the 48 Greek-owned reefers were at any time flagged to Greece; instead, 33 were flagged to Panama, 6 to Russia, and 3 each to Liberia and Vanuatu, and the remaining 3 were flagged to the Bahamas, Belize, and South Korea. The Chinese and Panamanian flags were the most common among the 16 reefers in the database for which owners could not be identified. Despite Panama’s significance as a flag state for the global reefer fleet, only one owner was based in Panama, operating a single reefer flagged to Togo (data file S1).

#### Reefer owner operations

**Fig. 3.**
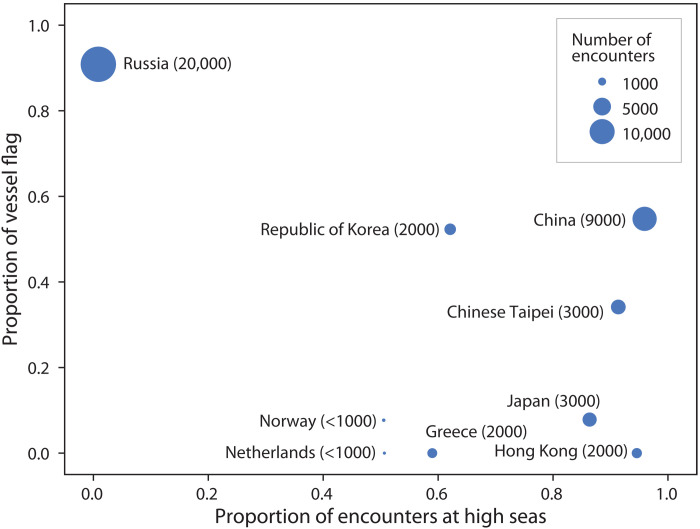
Reefer flags and operations on the high seas. For each owner’s country, we calculated the extent to which reefers are flagged to the same state where the owners are based (*y* axis) and the proportion of encounters taking place on the high seas (*x* axis). The dot size is proportional to the number of encounters (also indicated in parenthesis). Owner states with fewer than 10 vessels were excluded (data file S1).

By analyzing reefer activity on an annual basis, we found that the number of reefers engaged in transshipment stayed relatively stable over time, with ~300 reefers active each year. However, encounters between reefers and fishing vessels increased substantially from just over 5000 encounters in 2017 to more than 11,000 in 2022 (fig. S2). We also found that 66 of the 569 reefers were scrapped and taken out of service at some time during this study (data file S1). Our analysis of reefer owners’ activity based on the locations of fishing vessels encounters showed distinct patterns (Fig. 3). For instance, Russian-owned reefers primarily operated within the Russian exclusive economic zone (EEZ), whereas reefers owned by entities based in China, Chinese Taipei, Hong Kong SAR, and Japan were mainly active on the high seas. Chinese-owned reefers had the highest number of encounters and the highest number of reefers active on the high seas.

**Fig. 4.**
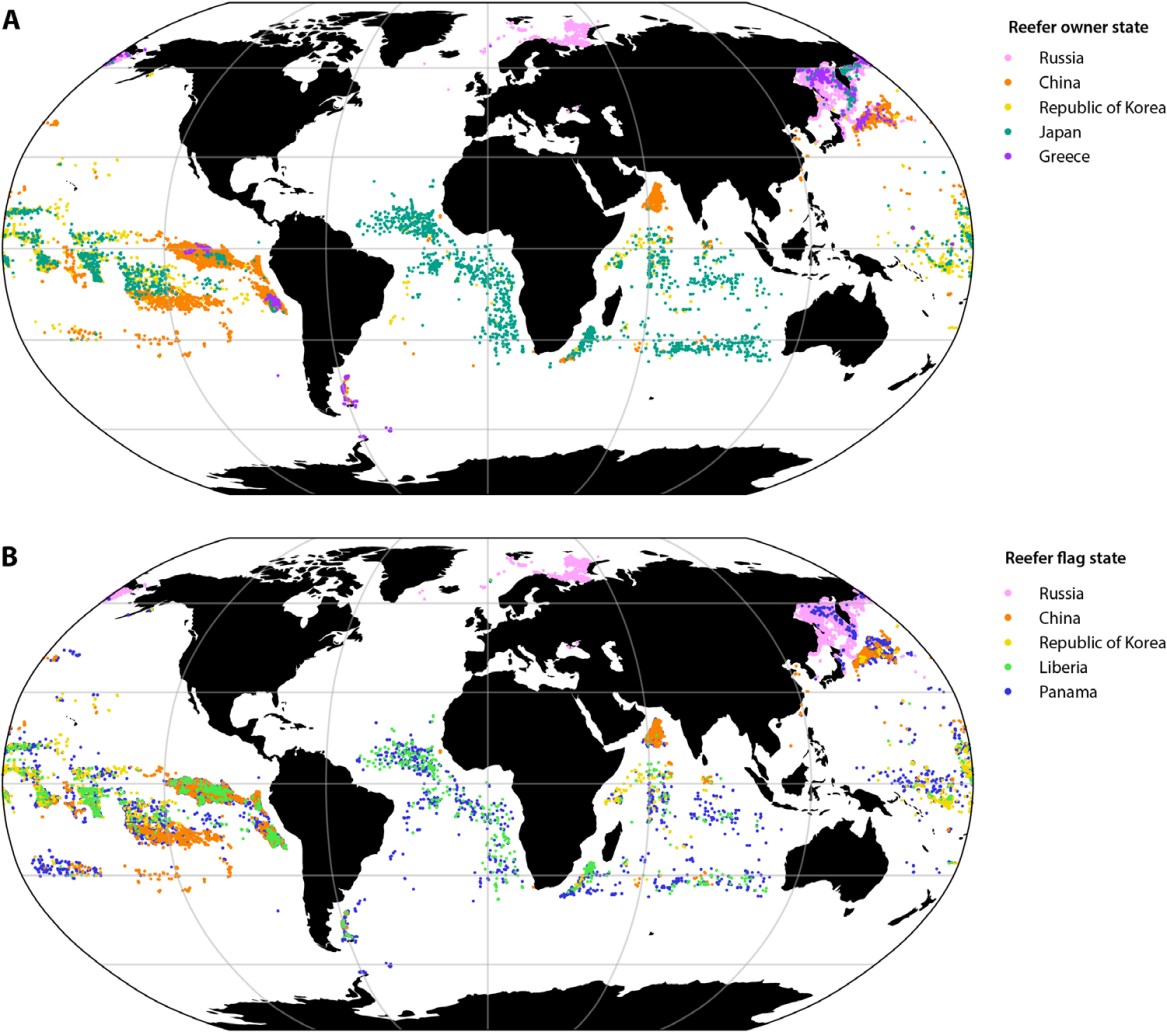
Who, where, and with what flag? (**A**) Encounters by country of ownership of the five largest reefer owners’ state. (**B**) Spatial footprint of the five most common reefer flags used. Explore an interactive version of the figure at https://stanfordcos.observablehq.cloud/reefer-analysis/.

Greek-owned reefers were primarily active in the Russian EEZ, particularly the Sea of Okhotsk, as well as on the high seas and in the Southern Ocean (Fig. 4A). Japanese-owned reefers dominated transshipment activity across the South Atlantic, especially along the Agulhas Current off South Africa, but were also active in the southern part of the Indian Ocean and across the Central Pacific Ocean. Chinese-owned reefers mainly operated in the Arabian Sea and off Ecuador, Peru, and Chile (especially to the north and west of Rapa Nui, with some activity in the North Pacific and off the coast of northern Japan). Owners based in South Korea mainly operated in the central and western Pacific Ocean, with additional activity off the coast of Somalia and into the Indian Ocean. In contrast, Russian owners primarily operated inside the Russian EEZ; in the North Atlantic off Norway, Svalbard, and Jan Mayen; and within Chinese and Korean waters (Fig. 4A).

Looking at reefer flags shows a different pattern (Fig. 4B). Russian-owned reefers predominantly use their domestic flag when operating within their EEZ, particularly in the North Atlantic off the coast of Norway and Russia. Reefers flagged to China, Panama, Liberia, and South Korea can be found in most oceans except the North Atlantic. Figure 4 (A and B) shows that Japanese-owned reefers operating in the South Atlantic were mainly flagged to Panama and Liberia. Chinese owners operating in the North Indian Ocean and South and North Pacific use the Chinese flag.

#### Owner interactions with fishing vessels’ flags and gears

**Fig. 5.**
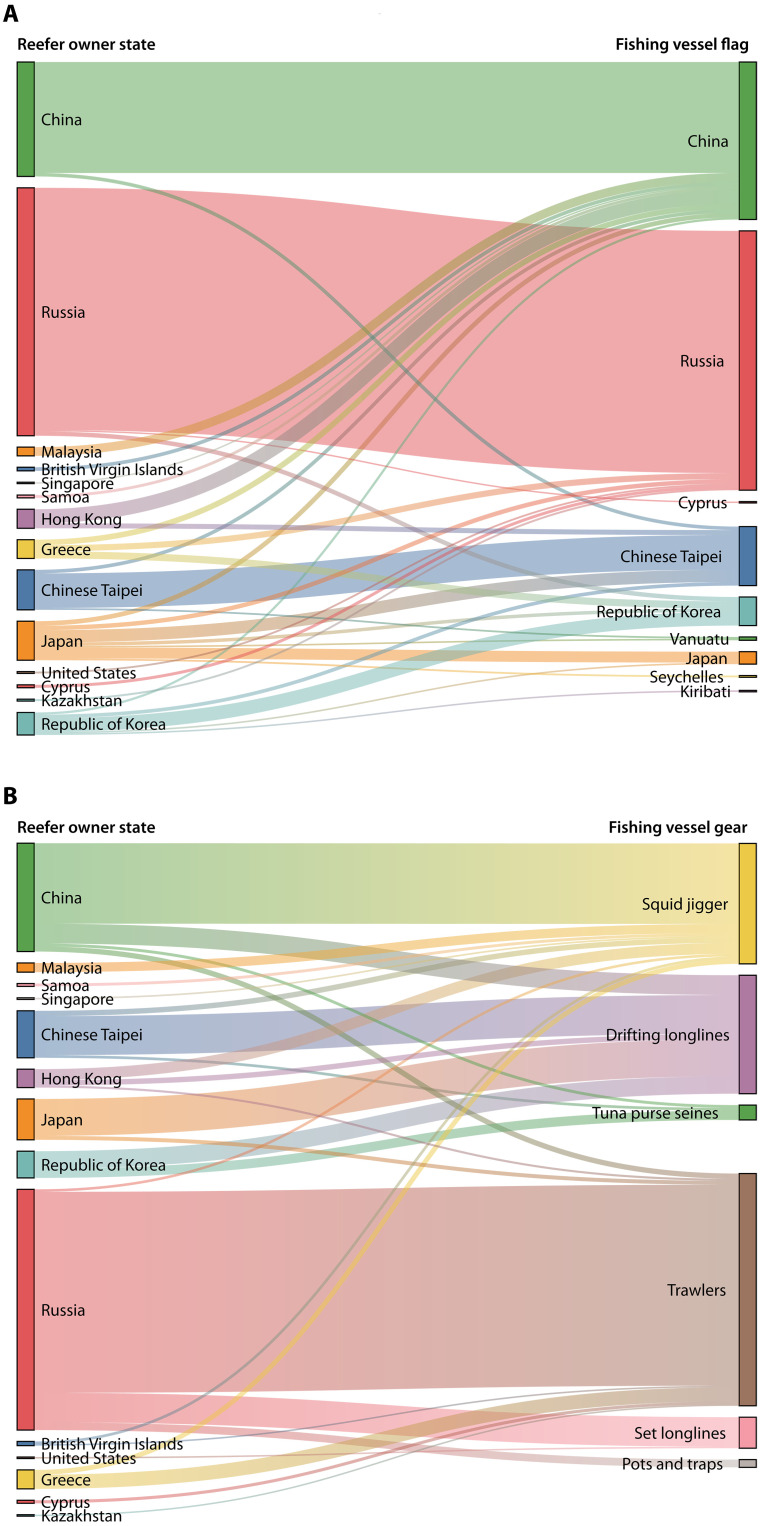
Encounters between reefers and fishing vessels. (**A**) Linkages between the country where the reefer owner is based and the flag of fishing vessels it encountered. (**B**) Linkages between the country where the reefer owner is based and the gear of fishing vessels that it encountered. Explore an interactive version of the figure at https://stanfordcos.observablehq.cloud/reefer-analysis/.

Between 2017 and 2022, reefers owned by entities in Russia, China, Chinese Taipei, and South Korea primarily met with fishing vessels flagged to their own states. In contrast, Greek and Japanese-owned reefers met with fishing vessels from diverse flags. Notably, Greek-owned reefers did not meet with any Greek-flagged vessels (Fig. 5A). Overall, the Greek company Laskaridis and the Korean company Green World Company Limited interacted with the widest diversity of fishing vessel flags, engaging with 323 fishing vessels flying 11 flags and 326 fishing vessels flying 10 flags, respectively. Despite this diversity, about half of the encounters by Korean owners were with Korean-flagged fishing vessels.

Our analyses suggest distinct patterns between the country of reefer ownership and the fishing gear of the fishing vessels encountered (Fig. 5B). We found that most Russian-owned reefers encountered trawlers. In contrast, Chinese-owned reefers primarily met with squid jiggers. Japanese- and Chinese Taipei-owned reefers had the highest proportions of encounters with drifting longliners, followed by Chinese and South Korean vessels (Fig. 5B). South Korean reefers also had the largest share of encounters with tuna purse seiners, followed by China and Chinese Taipei. Russian reefers had the largest share of encounters with set longliners and fishing vessels deploying pots and traps.

## DISCUSSION

Identifying vessels’ beneficial ownership, in particular, can be critical for determining who has ultimate legal responsibility and can be held accountable for potential illegal activities (*50*). Despite nascent efforts to identify ownership of fishing vessels (*51*), no study has looked at the ownership structure of the global reefer fleet, frequently described as an opaque activity with limited oversight. This study presents the first global overview of the owners of reefers, where they are based, links to specific geographical areas of operations, and the fishing vessel flags and types of fishing gear they encounter.

Our findings show that (i) a small number of states and owners are responsible for the majority of transshipment, with Russian and Chinese owners along with the Greek-based Laskaridis playing a pivotal role; (ii) reefers generally fly one of a few select flags, often corresponding to the flag of the fishing vessels with which they interact with, except for reefers owned by Greek and Japanese entities, which were found to interact with fishing vessels flying a wide diversity of flags; and (iii) multiple datasets must be considered to gain a clear understanding of the ownership structure and dynamics of reefers involved in fisheries. In the following sections, we discuss how our findings contribute to identifying additional leverage points for improved oversight and governance of transshipment.

### Ownership concentration

Concentration as a measure of companies’ impacts on the ocean and power to influence governance is increasingly used to understand the role of companies in the ocean economy (*51*–*53*). While previous studies have helped to interpret the scale and degree of consolidation of companies operating in the ocean economy (*52*, *53*), they have been critiqued for not clearly defining terms or using appropriate analytical tools to examine firms and their roles within value chains (*54*). Here, we use the number of reefer owners in each country as a unit to understand the reefer market and each owner’s role within their respective home states. In Greece and The Netherlands, a single company is responsible for virtually the entirety of reefer ownership in each country. By contrast, China and Russia are home to many reefer owners, each accounting for a comparatively small portion of each country’s reefer ownership. Therefore, we show that national dynamics can be quasi-monopolistic or highly competitive within what is described as a globally “closed” reefer market (*48*, *55*). The higher HHI value for Greece and The Netherlands indicates stronger market concentration, with fewer firms dominating the market and less competition. Conversely, a lower HHI value, such as for China and Russia, indicates a more competitive market with many firms each having smaller shares of the market.

### Reefer owners’ operations

Transshipment operations related to Russia and China are particularly interesting due to the size of their fleets and the scale of their operations (*28*). Previous studies on transshipment, such as those by Petrossian *et al.* (*33*) and Greenpeace International (*56*), excluded Russian interactions because most of these interactions occur between Russian-owned and Russian-flagged reefers and Russian fishing vessels within Russian-controlled fisheries, forming a self-contained system. Yet, cod, haddock, and pollock caught in Russian fisheries enter the global market and are part of the global seafood supply chain. By focusing on reefer owners and including Russia in our analysis, we have linked owners such as the Greek-based Laskaridis to operations in Russia, demonstrating the value of examining ownership alongside flags and operational activities to understand transshipment better. This focus on owners is also beneficial in the South Atlantic, where reefer activity in the eastern part appears dominated by a small number of Japanese owners (data file S1). Given the limited number of owners (324) identified in this study, the industry concentration, and the spatial extent of operations, our findings support the argument that transshipment can be understood as a specialized activity (*48*, *55*, *57*) with discernible patterns. The information provided here and the accompanying database can be used for targeted conversations and interventions aimed at specific owners or groups of owners in particular regions to support sustainability and transparency.

### Why does the owner’s choice of flag matter?

While Russian authorities regulate transshipments in Russian waters in their dual role as coastal and flag states, conditions are more complicated for other authorities. In the case of China, for instance, Chinese owners with reefers flagged to China or with a foreign flag primarily interact with Chinese-flagged fishing vessels outside China’s EEZ and, in particular, squid fisheries with and without agreed management rules. Still, when Chinese-flagged fishing vessels fish on the high seas in areas without agreed management measures, China, as any flag state, is obligated to cooperate with relevant international organizations for the protection and preservation of the marine environment in line with the UN Convention of Law of the Sea (*47*, *58*).

For owners of reefers providing services to fisheries managed by RFMOs, the flag that they fly matters. A vessel’s flag is essential because it determines which RFMO it can operate within and which ports it can visit. The different efforts undertaken by RFMOs in managing transshipment activities and the need to harmonize CMMs across RFMOs have been the focus of several studies (*22*, *59*). Panama, a flag state for 189 of the 569 reefers in our database, with combined operations that span across the ocean and multiple fisheries and RFMOs, is only directly engaged in decision-making in 6 of the 17 RFMOs globally (*60*). More robust engagement from Panama and the owners of their flagged vessels across the RFMOs that they operate could foster valuable discussions on the global adoption of guidelines, harmonization of rules, and swift implementation of best practices across RMFOs.

Reefer owners are increasingly faced with additional requirements, such as the European Union (EU) Competent Authority Authorization, which only allows cargo from vessels flagged to certain preapproved countries to be traded within the EU (*61*). Authorization from the EU Competent Authority has become an essential consideration in the choice of flag state for reefers, particularly for seafood used in the European canning sector. Consequently, 16 reefers previously flagged to Liberia and Vanuatu were reflagged to States with EU Competent Authority authorization (*62*, *63*). Recently, Russian-flagged reefers were denied entry into ports in The Netherlands due to the war in Ukraine and concerns about Russian-flagged vessels being used for espionage. This led the Russian seafood company Norebo to contract a Norwegian-owned and Norwegian-flagged reefer (IMO 9143386) to transship fish from the Barents Sea destined for markets outside Russia (*64*).

### Engaging reefer owners for increased governance and transparency

**Fig. 6.**
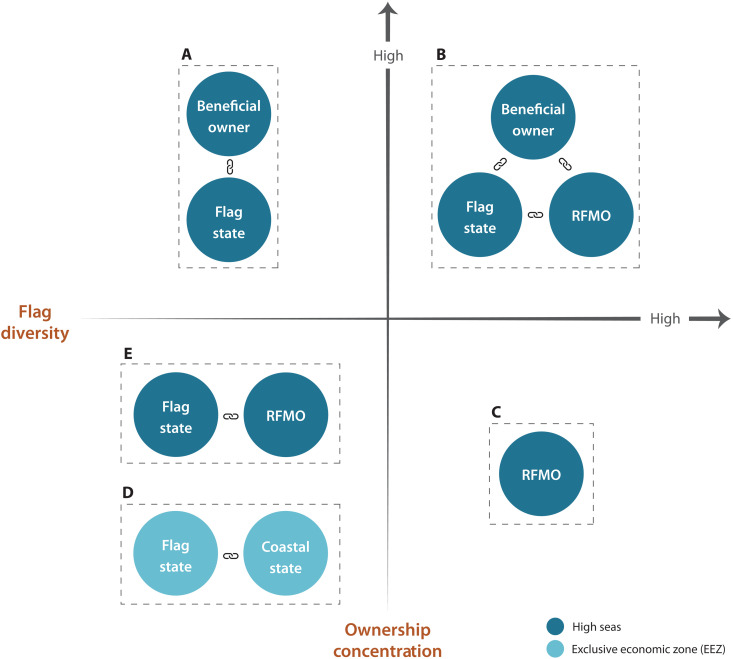
Conceptual figure illustrating interactions of relevance for transshipment governance according to reefer flag diversity and ownership concentration. (**A** to **E**) The scenarios are drawn from interactions identified in our analyses. Key actors considered to support improved governance include beneficial owners, flag states, coastal states, and RFMOs.

Our study has identified previously unidentified leverage points for transshipment reform by identifying reefer owners and the various geographical and regulatory contexts that they operate within. Exploring these leverage points and linking them to relevant national and international interventions can contribute to greater transparency and increase accountability (Fig. 6).

In situations with a high concentration of owners and low diversity of flags, working directly with those owners or encouraging public-private partnerships (PPP) between owners and states governing the flags or fishing areas (including through RFMO membership) could provide leverage. This approach could be relevant inside the Commission for the Conservation of Antarctic Marine Living Resources (CCAMLR), where the majority of reefers operating are owned by just two companies, Laskaridis and Aker ASA, and fly a few select flags (https://stanfordcos.observablehq.cloud/reefer-analysis/). Paired with the Association of Responsible Krill Harvesters (ARK) commitment to improve transshipment oversight inside CCAMLR (*65*), a PPP between reefer owners and fishing companies could enhance transshipment management and oversight. This is especially relevant because CCAMLR members have repeatedly failed to agree on revised CMMs for transshipment (Fig 6A).

In situations with a high concentration of owners and flag diversity, PPPs can enhance governance through stronger collaborations inside the RFMO. This approach may be appropriate within the Indian Ocean Tuna Commission (Fig 6B).

Where ownership concentration is low and the diversity of flags is high, the contributions of PPPs may be lower and less effective for enhancing governance. This pattern is evident in the Inter-American Tropical Tuna Commission (IATTC) and the Western and Central Pacific Fisheries Commission (WCPFC) (Fig 6C). In these cases, continued efforts to improve RFMO management are crucial. In addition, in instances of low ownership concentration and high flag diversity, precompetitive collaborations such as ARK or industry roundtables between owners of key carriers and networks, as identified by Petrossian *et al.* (*33*), could provide additional mechanisms for improved management and transparency.

For transshipment operations carried out in the Russian EEZ (Fig. 6D) or by Chinese owners (Fig. 6E) in international waters, where there is low ownership concentration and low flag diversity, the flag state will be of vital importance. These flag states must adhere to coastal state regulations and collaborate with RMFOs where they exist. For instance, Russian-flagged fishing vessels transship catches in the North-East Atlantic cod and haddock fishery, regulated by the North-East Atlantic Fisheries Commission (NEAFC). These transshipments are subject to NEAFC port state measures, established in 2007 (*1*, *19*) and later updated in alignment with the Port State Measures Agreement (PSMA), which entered into force in 2016. The PSMA seeks to prevent IUU fishing catches from entering global markets by requiring minimum standards on inspection measures when foreign vessels seek access to the ports of PSMA parties and by denying entry to vessels involved in IUU fishing (*19*).

### The data and information sharing challenge

Accessing and using AIS data helps inform science, policy, and public debate. Still, vessel data alone is often insufficient to identify all available leverage points (*36*, *66*, *67*). We show that combining vessel data with ownership information can provide essential insights into transshipment practices. This study relies heavily on information already collected and publicly available to varying degrees but poorly shared among relevant authorities and organizations (*43*, *68*). Improving data accessibility will require increased collaboration between the organizations that manage reefers (e.g., IMO) and regulatory bodies for fisheries (e.g., RFMOs). National fishery managers and RFMOs can promote greater transparency by ensuring reefers authorized to operate within their jurisdiction share all relevant information on vessel ownership. A step change in transparency could be achieved by making it mandatory to provide publicly available information about beneficial owners as part of any licensing processes (*50*). Reefer owners could also engage with their respective flag states to support the swift and full implementation of the Global Record of Fishing Vessels, Refrigerated Transport Vessels and Supply Vessels (Global Record), an initiative hosted by FAO, which aims to enhance transparency and traceability by providing a single access point for all vessels used in fishing.

IMO also provides publicly accessible information on owners through the Company and Registered Owner Identification Number Scheme, introduced in 2004. This scheme applies to each company or registered owner managing ships of 100 gross tonnage and above engaged in international voyages (*69*), a definition which includes most reefers. The measure was adopted to enhance maritime safety, security, and environmental protection and to prevent maritime fraud. Managed in parallel with a vessel’s identification number scheme, it could enable better oversight of owners if integrated into the Global Record. This kind of data sharing is a key aspiration of the PSMA and could be advanced by its rapid and full implementation.

Identifying beneficial owners involved in transshipment generates opportunities for novel approaches to engagement. The information described in this work and our publicly available database can be used by scientists, civil society groups, governments, international organizations, financial institutions, and other stakeholders to identify relevant corporate actors to assist in implementing recently published global guidelines on transshipment (*14*), industry-led voluntary commitments, and best practices across fisheries. Requiring more transparent ownership information could aid shipping insurers and financiers pressuring vessel owners to uphold higher standards (*70*). All stakeholders should be vested in finding effective ways to monitor the reefer fleet with greater transparency to ensure full compliance with legal and sustainability standards in fisheries. Full legality is not only in the interest of the majority but also a legal requirement for any flag state.

## MATERIALS AND METHODS

### Definition of reefers

We applied a definition of reefers from the shipping literature describing reefers as the smallest vessels involved in global cargo with the distinct feature of transporting chilled and frozen goods and having their cranes/derricks, allowing them to operate at sea or in smaller ports (*18*, *48*). Goods are mostly loaded as pallets or loose goods through hatches on top of the vessel by engaging in ship-to-ship transfer operations. Non-reefers such as well-boats primarily used in aquaculture, tugs, fish factories, bunker vessels, fishing vessels, and local cargo vessels were removed from the data accessed through the Carrier Vessel Portal (section S2). All remaining reefers were manually checked across multiple datasets to confirm their type (Table 1).

**Table 1.** Primary sources of data used to build and analyze the reefer database. Data were collected until the end of January 2023 to cover the entire year of 2022. NPFC, North Pacific Fisheries Commission; ICCAT, International Commission for the Conservation of Atlantic Tunas; SPRFMO, South Pacific Regional Fisheries Management Organization.

Database	Description	Link
Global Fishing Watch (GFW) Carrier Vessel Portal	Data are provided and curated by the NGO GFW. The portal uses publicly available data from 2017 to present with a 72-hour delay to identify potential vessel encounters. Updated monthly.	https://globalfishingwatch.org/carrier-vessel-portal/
		(Public but requires login)
IMO’s Global Integrated Shipping Information System	Data hub for the global shipping industry. Developed and maintained by IMO.	https://gisis.imo.org/Public/Default.aspx
		(Public but requires login)
Lloyd’s Seasearcher	Lloyds has a 300-year history in providing shipping news and intelligence and is a world leading provider of such information. It is a subscription-based service that collects detailed information on vessels, companies, places, incidents, and sanctions and also provides analyses on the maritime industry.	www.seasearcher.com/
		(Accessible via paid subscription)
Russian Maritime Register of Shipping	The Russian Maritime Register of shipping is a classification society dating back to 1913.	https://rs-class.org/en/
		(Public)
RFMO vessel registries	RFMOs keep a list of vessels authorized to conduct activities in their regulatory area.	NPFC, CCAMLR, WCPFC, ICCAT, and SPRFMO
		www.sprfmo.org/web/public/vessel
		https://vessels.wcpfc.int
		www.npfc.int/compliance/vessels
		www.ccamlr.org/en/compliance/list-authorised-vessels
		(Not all RFMO lists are publicly available from their respective websites.)
Ship spotting and ship information sites	Sites open to the public to conduct searches on specific vessels or areas.	www.marinetraffic.com
		www.balticshipping.com/
		www.shipspotting.com

### Data sources and cleaning

This paper analyzes reefers based on multiple datasets downloaded from Global Fishing Watch (GFW) Carrier Vessel Portal between 2017 and 2022. The years 2017 to 2022 were selected because of the poor availability of satellite data before 2017. The Carrier Vessel Portal provides the only publicly accessible AIS-based dataset of what GFW defines as carrier vessels the fishing industry uses (see section S1 for GFW definitions). The portal is a living database with a continuously evolving number of vessels added to the database. As of January 2023, it included a total of 868 carrier vessels. We retained all identified reefers that had been active from 2017 to 2022, including 81 vessels that were identified as reefers but did not encounter fishing vessels between 2017 and 2022 (section S2).

### MMSI and vessel IMO number

The data provided via the download function of the Carrier Vessel Portal consisted of vessels’ MMSI numbers, flags, and encounters but lacked the IMO number or information related to ownership. A vessel’s IMO number is a unique identifier, mandatory since January 1996, that stays the same throughout a vessel’s life span. The IMO number is attached to the hull of the ship (*40*). For each reefer, we added the vessel’s unique IMO number verified using the Lloyd’s List Intelligence Seasearcher database, the IMO’s Global Integrated Shipping Information System database, RFMO records, and national registries like the Russian Maritime Register of Shipping, as well as visual observations based on images from ship spotting sites (Table 1). All MMSI numbers associated with a particular reefer were kept in the database to track ownership and flag information for each reefer over time. The same reefer can, therefore, appear multiple times with the same IMO number but with different MMSI, flag, and/or ownership and operators (data file S1).

### Definition of beneficial owners and operators

Beneficial owners are the ones who ultimately decide how their reefers will be used and retain liability for them, even if the activity is carried out without their knowledge or consent. Owners are also the ultimate beneficiaries of revenue generated by the vessels they own (*46*, *71*). For this work, we used the definition applied by Lloyd’s List Intelligence (hereafter “Lloyds”) to determine what constitutes a beneficial owner: “the ultimate owning entity, controlling party or representative thereof which can be an individual, company group or organization.” Information on the country where the owners are based is determined by the location of the beneficial owner’s headquarters. It is not uncommon for the vessel’s “registered owner” to be a subsidiary of the “beneficial owner.” A ship’s registered owner is the company or individual to whom the ship’s legal title of ownership has been registered in a ship’s registry. A study of global ship-owning showed that in 2020, 90% of registered owners were so-called “one-ship companies,” indicating that entities legally owning more than one ship are rare (*72*). For this study, we focused on the beneficial owners as they are the ultimate legal entity of a vessel and not the registered owner because the registered owner, in many cases, is a corporation without any assets other than the ship itself. For 16 reefers in our database, we could not find the beneficial owners but did locate data on flags and links to registered owners as the vessels are kept in the database. A third party may also operate a ship, called an operator, who can be the vessel owner or a company operating a vessel on behalf of others. A ship can also be chartered to a shipper, time chartered, or chartered for a specific voyage (*73*). Lloyds define the operator as “responsible for the commercial direction of a ship, including its employment, and that the Commercial Operator may be the principal operating affiliate of the Beneficial Owner.” Reefers can also be used in a profit-sharing scheme by a group of reefer owners called a reefer pool (Box 2), where all vessels inside the pool share revenues (*48*, *49*). A beneficial owner can have vessels in different pools, with smaller reefers for fish in one and larger reefers suitable for fruits in another.

Many reefers are also registered by their owners in so-called open registries. An open ships registry, also known as a flag of convenience, is a system where ship owners can register their vessels in a country other than their own to take advantage of more lax regulations and tax on profits of trading ships is low or absent, or requirements concerning maintenance or manning might be more relaxed. The two largest ship registries in tonnage, Libera and Panama, are open registries.

### Hirschman-Herfindahl index

To understand the market concentration of the owners in each country in relation to the reefer industry as a whole, we calculated the HHI. A higher HHI score means that the sector is more concentrated, while a lower score means that the industry is more competitive. We selected the number of reefers belonging to each owner as a proxy for market share.

### Limitations

The data used in this research rely heavily on definitions used and data provided by third parties, such as GFW and Lloyd’s Seasearcher. When possible, additional data from open sources such as ship spotting sites and IMO have been used to triangulate and increase data accuracy. Ships are assets that can be sold, traded, and reflagged at any time which allows for historical records but can present current inaccuracies. An additional limitation is that Lloyd’s Seasearcher is a subscription service limiting the verification of the data provided in our database. To the best of our ability and with the resources available, we have tried to find those companies that own reefers used by the fishing industry. We hope this information will become more accessible and see this paper and the accompanying database as an essential step.

## Supplementary Materials

### The PDF file includes:

Sections S1 and S2 Figs. S1 and S2 Legend for data file S1 References
