# Supplementary material for: Who owns reefer vessels? Uncovering the ecosystem of transshipment in fisheries
Source: Sci Adv. 2024 Oct 11;10(41):eadn3874. doi: 10.1126/sciadv.adn3874 (PMC11480961; doi:10.1126/sciadv.adn3874)
Supplement: Supplementary file 1 — Sections S1 and S2 Figs. S1 and S2 Legend for data file S1 References [file sciadv.adn3874_sm.pdf]

Science Advances
AAAS

Supplementary Materials for

Who owns reefer vessels? Uncovering the ecosystem of transshipment in fisheries

Frida Bengtsson *et al.*

Corresponding author: Frida Bengtsson, frida.bengtsson@su.se

Sci. Adv. **10**, eadn3874 (2024)
DOI: 10.1126/sciadv.adn3874

The PDF file includes:

Sections S1 and S2
Figs. S1 and S2
Legend for data file S1
References

Other Supplementary Material for this manuscript includes the following:

Data file S1

Supplementary Text

Section S1. Definitions from Global Fishing Watch of relevance for this work.

Carrier vessel: A carrier vessel is a type of vessel used to transport fish, supplies, and crew from fishing vessels, allowing fishing vessels to stay out at sea for extended periods. A carrier vessel can receive catch, or ´tranship´, from one or more fishing vessels at sea or in port and then offload the catch at a chosen port or fish processing location (https://globalfishingwatch.org/faqs/what-is-a-carrier-vessel/)

Encounter: An encounter is anywhere that a refrigerated cargo vessel meets up with a fishing vessel while at sea. Encounters may indicate potential transshipment activity between two vessels that both appear in the automatic identification system (AIS) data source. Our algorithm estimates an encounter event when two vessels are within 500 meters for at least two hours and travelling at a median speed < two knots while at least 10 km from a coastal anchorage (https://globalfishingwatch.org/faqs/what-is-a-vessel-encounter/)

Loitering: Loitering is when a single vessel exhibits behaviour indicative of a potential encounter event. Loitering is estimated using automatic identification system (AIS) data, including vessel speed, duration in a given location, and distance from shore. Loitering occurs when a vessel travels at an average speed of < two knots while at least an average of 20 nautical miles from shore. To reduce noise in the carrier vessel portal, only loitering events greater than one hour are shown (https://globalfishingwatch.org/faqs/what-is-loitering-event/)

Section S2. Additional information on the types of vessels included or excluded in the database.

Reefers without encounters:

There could be multiple explanations for why these reefers had no encounters with fishing vessels. For instance, the flag state, RFMO member or fishing company may have notified relevant authorities of a future intention to conduct transshipments, and the vessel has subsequently been added to authorised vessel lists provided by Regional Fisheries Management Organizations (RFMOs). These vessel lists are regularly matched with the Carrier Vessel Portal. The reefers may also have displayed patterns of loitering as defined by Global Fishing Watch and met a fishing vessel, but the event could not be verified based on public records (section S1). One such reefer is the Kenta Maru (IMO 9788772), owned by Toei Reefer Line, which has not been recorded as encountering any fishing vessels according to the data in the Carrier Vessel Portal used for the analysis but is authorised to tranship on the High Seas by multiple RFMOs such as the Western and Central Pacific Fisheries Commission (WCPFC) (https://vessels.wcpfc.int/vessel/11656 accessed on 12.04.2023).

Broken up vessels

While searching for owners of reefers, we came across multiple reefers displayed at having the status *dead* in Lloyd's Seasearcher and with known places for ship breaking, e.g., Alang in India or Chittagong in Bangladesh as their last known destination and/or with a transfer of ownership to owners such as Indian Breakers or Turkish Breakers. By assembling public lists of vessels broken up and kept by the NGO Shipbreaking platform, we found an annual average of 11 reefers decommissioned between 2017 and 2022. We identified 66 reefers in our database that had been broken up during the period used in this research. The large number of broken-up reefers can be linked to the fleet's average age of 29 years (*18*). Ship owners call the reefer trade a business in a run-off mode and a niche market serving particular trades (*55*).

The activity of a small number of fish factories

We identified 16 fish factories or motherships in the data, 4 of which had been dismantled, leaving 12 vessels in operation. Nine of the 12 vessels in operation were flagged to Russia, two to the United States and one to Norway. The extensive use of large motherships for seafood processing was developed after World War II, and some of the largest vessels were operated by the Soviet Union (*77*) These vessels receive catches from other vessels for onboard processing, such as canning. The largest fish processing vessel today is the Vladivostok 2000 (IMO 7913622 and current MMSI 273455520). The vessel has had 7 different names and 11 beneficial owners, and it has changed its flag 12 times since it was built in 1980, according to Lloyds Seasearcher.

Well-boats used in wild-capture fisheries

Different well-boats have historically been used to keep wild-caught fish alive and transported to markets (*78, 79*). Well-boats are also used in Norwegian small-scale coastal pelagic fishing since smaller fishing vessels can't transport their catches to buyers. Fishing vessels lock their catches, and pre-approved well-boats with certified scales assist these fisheries with transport. Norwegian regulations regulate this activity, which is not classified as a transshipment (*80-82*). Well-boats have also been found to operate along the coast of Chile near large aquaculture operations. Well-boats were excluded from the database as the technical specifications and regulations differ from reefers, even if they can belong to a broader definition of fish carriers or fisheries support vessels.

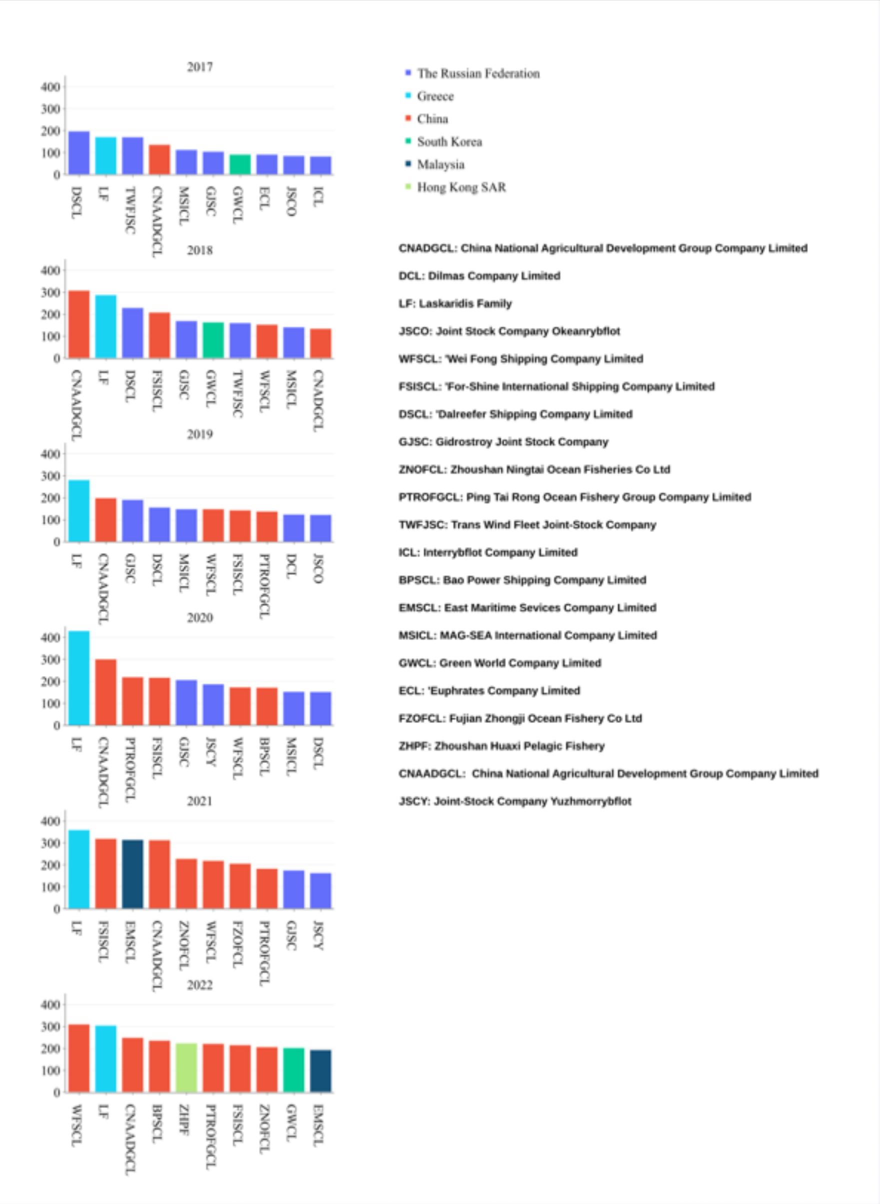

Fig. S1. The ten most active reefer owners based on the cumulative number of encounters between reefers and fishing vessels for 2017-2022.

The figure shows the owners with the most encounters with fishing vessels based on the number of encounters only. The number of encounters could indicate the size of operations and relative importance.

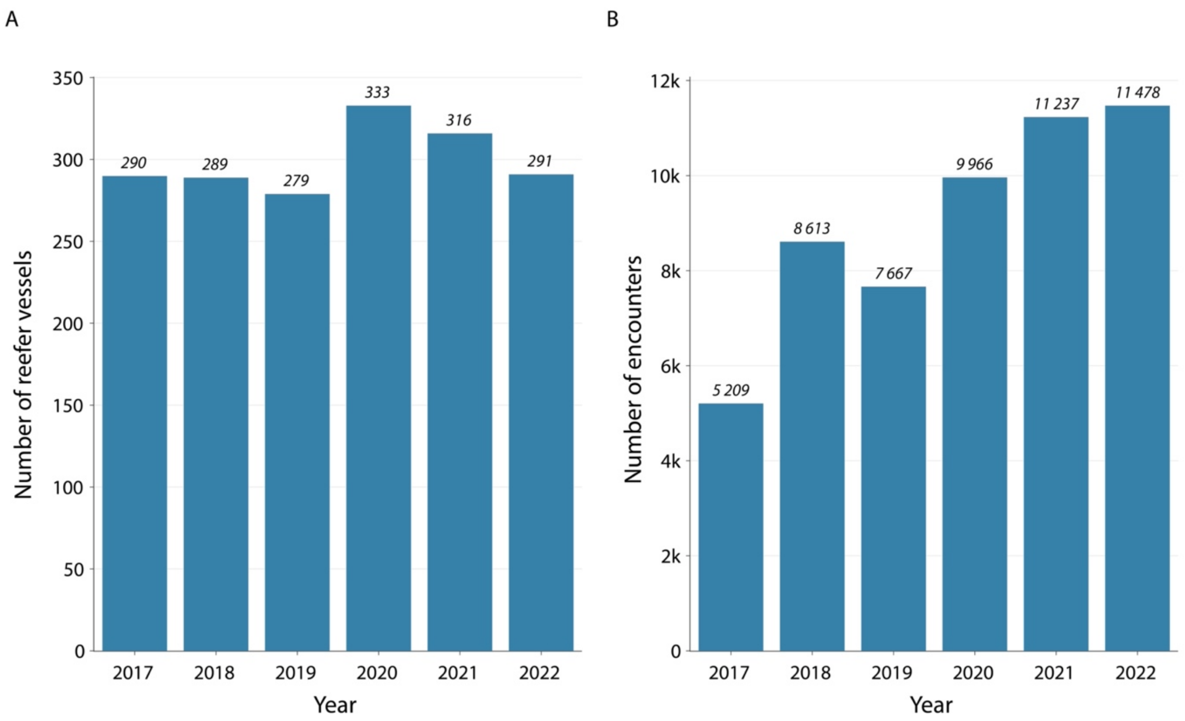

Fig. S2. Numbers of reefers and encounters. (**A**) The total number of reefers (based on reefers' unique IMO number) and (**B**) encounter events between reefers and fishing vessels for the years 2017-2022. While the number of encounters has increased, the number of reefers has stayed relatively stable. An encounter, as defined by Global Fishing Watch (GFW), is an event during which a refrigerated cargo vessel is within 500 meters of a fishing vessel for at least two hours, travelling at a median speed of less than two knots, while at least 10 km from a coastal anchorage (section S1).

Data file S1. Vessels, owners, operators and flags (Excel file).

REFERENCES AND NOTES

1. O. S. Stokke, Trade measures and the combat of IUU fishing: Institutional interplay and effective governance in the Northeast Atlantic. *Mar. Policy* **33**, 339–349 (2009).

2. Food and Agricultural Organization (FAO), *International Plan of Action to Prevent, Deter and Eliminate Illegal, Unreported and Unregulated Fishing* (FAO, 2001).

3. High Seas Task Force, *Closing the Net: Stopping Illegal Fishing on the High Seas* (High Seas Task Force, 2006).

4. R. Blasiak, A. Dauriach, J.-B. Jouffray, C. Folke, H. Österblom, J. Bebbington, F. Bengtsson, A. Causevic, B. Geerts, W. Grønbrekk, Evolving perspectives of stewardship in the seafood industry. *Front. Mar. Sci.* **8**, 676 (2021).

5. D. J. Agnew, J. Pearce, G. Pramod, T. Peatman, R. Watson, J. R. Beddington, T. J. Pitcher, Estimating the worldwide extent of illegal fishing. *PLOS ONE* **4**, e4570 (2009).

6. B. Le Gallic, A. Cox, An economic analysis of illegal, unreported and unregulated (IUU) fishing: Key drivers and possible solutions. *Mar. Policy* **30**, 689–695 (2006).

7. A. Leroy, F. Galletti, C. Chaboud, The EU restrictive trade measures against IUU fishing. *Mar. Policy* **64**, 82–90 (2016).

8. B. Soyer, G. Leloudas, D. Miller, Tackling IUU fishing: Developing a holistic legal response. *Transnat. Environ. Law* **7**, 139–163 (2018).

9. M. A. Young, International trade law compatibility of market-related measures to combat illegal, unreported and unregulated (IUU) fishing. *Mar. Policy* **69**, 209–219 (2016).

10. Food and Agricultural Organization (FAO), *Agreement on Port State Measures to Prevent, Deter and Eliminate Illegal Unreported and Unregulated Fishing* (FAO, 2016).

11. U. R. Sumaila, J. Alder, H. Keith, Global scope and economics of illegal fishing. *Mar. Policy* **30**, 696–703 (2006).

12. A. M. Cabanelas, *Transshipment: A Closer Look: An in-Depth Study in Support of the Development of International Guidelines* (FAO, 2020).

13. The Pew Charitable Trusts (PEW), *Transshipment Reform Needed to Ensure; Legal, Verifiable Transfer of Catch* (PEW, 2018).

14. Food and Agricultural Organization (FAO), *Voluntary Guidelines for Transshipment / Directives Volontaires Relatives au Transbordement / Directrices Voluntarias para los Transbordos* (FAO, 2023).

15. D. Tickler, J. J. Meeuwig, M.-L. Palomares, D. Pauly, D. Zeller, Far from home: Distance patterns of global fishing fleets. *Sci. Adv.* **4**, eaar3279 (2018).

16. W. Swartz, E. Sala, S. Tracey, R. Watson, D. Pauly, The spatial expansion and ecological footprint of fisheries (1950 to present). *PLOS ONE* **5**, e15143 (2010).

17. Western and Central Pacific Fisheries Commission, CMM 2009-06 - Conservation and Management Measures on the Regulation of Transhipment (Western and Central Pacific Fisheries Commission, 2010); https://cmm.wcpfc.int/measure/cmm-2009-06.

18. Dynamar, Reefer Analysis 2021: Market Structure, Conventional, containers (Dynamar, 2021).

19. LRQA, Barents Sea cod, haddock and saithe (Marine Stewardship Council fisheries assessments, LRQA, 2022).

20. Intertek Moody Marine, Russian Sea of Okhotsk Mid-water Trawl Walleye Pollock (Theragra chalcogramma) Fishery (Public Certification Report, Derby, 2013).

21. United Nations Fish Stocks Agreement (UNFSA), The United Nations Agreement for the Implementation of the Provisions of the United Nations Convention on the Law of the Sea of 10 December 1982 Relating to the Conservation and Management of Straddling Fish Stocks and Highly Migratory Fish Stocks (in Force as from 11 December 2001) (U.N. DOCA/Conf. 164/37, UNFSA, 1995); www.un.org/depts/los/convention_agreements/texts/fish_stocks_agreement/CONF164_37.htm.

22. C. Ewell, S. Cullis-Suzuki, M. Ediger, J. Hocevar, D. Miller, J. Jacquet, Potential ecological and social benefits of a moratorium on transshipment on the high seas. *Mar. Policy* **81**, 293–300 (2017).

23. C. Bueger, T. Edmunds, Blue crime: Conceptualising transnational organised crime at sea. *Mar. Policy* **119**, 104067 (2020).

24. United Nations Office on Drugs and Crime (UNODC), *Fisheries Crime* (UNODC, 2021).

25. G. Bichler, G. A. Petrossian, K. Viramontes, N. Marteache, Detecting communities at high-risk of IUU fishing: Networks of shadow encounters in Area 81 of the Western Central Pacific. *Front. Mar. Sci.* **11**, 1355481 (2024).

26. D. D. Miller, U. R. Sumaila, D. Copeland, D. Zeller, B. Soyer, T. Nikaki, G. Leloudas, S. T. Fjellberg, R. Singleton, D. Pauly, Cutting a lifeline to maritime crime: Marine insurance and IUU fishing. *Front. Ecol. Environ.* **14**, 357–362 (2016).

27. L. Malarky, B. Lowell, No more hiding at sea: Transshipping exposed (Oceana, 2017).

28. N. A. Miller, A. Roan, T. Hochberg, J. Amos, D. A. Kroodsma, Identifying global patterns of transshipment behavior. *Front. Mar. Sci.* **5**, 240 (2018).

29. E. R. Selig, S. Nakayama, C. C. C. Wabnitz, H. Österblom, J. Spijkers, N. A. Miller, J. Bebbington, J. L. Decker Sparks, Revealing global risks of labor abuse and illegal, unreported, and unregulated fishing. *Nat. Commun.* **13**, 1612 (2022).

30. Food and Agricultural Organization (FAO), Global Study on Transshipment, Regulations, Practices, Monitoring and Control (FAO, 2018).

31. J. L. Decker Sparks, L. K. Hasche, Complex linkages between forced labor slavery and environmental decline in marine fisheries. *J. Hum. Rights* **18**, 230–245 (2019).

32. D. Belhabib, P. Le Billon, Fish crimes in the global oceans. *Sci. Adv.* **8**, eabj1927 (2022).

33. G. A. Petrossian, B. Barthuly, M. C. Sosnowski, Identifying central carriers and detecting key communities within the global fish transshipment networks. *Front. Marine Sci.* **346**, 798893 (2022).

34. K. Seto, N. Miller, M. Young, Q. Hanich, Toward transparent governance of transboundary fisheries: The case of Pacific tuna transshipment. *Mar. Policy* **136**, 104200 (2022).

35. IMO, *Resolution A.1106(29) Revised Guidelines for the Onboard Operational Use of Shipbourne Automatic Identification Systems (AIS)* (2015).

36. Windward, Mind the AIS gap (2019); https://windward.ai/blog/mind-the-ais-gap/.

37. J. L. Shepperson, N. T. Hintzen, C. L. Szostek, E. Bell, L. G. Murray, M. J. Kaiser, A comparison of VMS and AIS data: The effect of data coverage and vessel position recording frequency on estimates of fishing footprints. *ICES J. Mar. Sci.* **75**, 988–998 (2018).

38. D. J. McCauley, P. Woods, B. Sullivan, B. Bergman, C. Jablonicky, A. Roan, M. Hirshfield, K. Boerder, B. Worm, Ending hide and seek at sea. *Science* **351**, 1148–1150 (2016).

39. K. Boerder, N. A. Miller, B. Worm, Global hot spots of transshipment of fish catch at sea. *Sci. Adv.* **4**, eaat7159 (2018).

40. IMO, *Resolution A.1117(30)* (2017).

41. International Telecommunication Union, M.585: Assignment and use of identities in the maritime mobile service (2022); www.itu.int/rec/R-REC-M.585-9-202205-I/en.

42. L. Drakopulos, J. J. Silver, E. Nost, N. Gray, R. Hawkins, Making global oceans governance in/visible with Smart Earth: The case of Global Fishing Watch. *Environ. Plan. E: Nat. Space* **6**, 1098–1113 (2022).

43. J. Park, J. Van Osdel, J. Turner, C. M. Farthing, N. A. Miller, H. L. Linder, G. Ortuño Crespo, G. Carmine, D. A. Kroodsma, Tracking elusive and shifting identities of the global fishing fleet. *Sci. Adv.* **9**, eabp8200 (2023).

44. G. G. McDonald, C. Costello, J. Bone, R. B. Cabral, V. Farabee, T. Hochberg, D. Kroodsma, T. Mangin, K. C. Meng, O. Zahn, Satellites can reveal global extent of forced labor in the world's fishing fleet. *Proc. Natl. Acad. Sci.* **118**, e2016238117 (2021).

45. B. N. Metaxas, *Flags of Convenience and Shipping Strategies, Maritime Research and European Shipping and Shipbuilding* (Netherlands Maritime Institute, 1978).

46. H. Griffiths, M. Jenks, Headline data: Vessel owners, types and age (Stockholm International Peace Research Institute, 2012).

47. UNCLOS, *United Nations Convention on the Law of the Sea* (UNCLOS, 1982).

48. T. Taro Lennerfors, P. Birch, *Snow in the Tropics: A History of the Independent Reefer Operators*, (Brill Publishers, 2019).

49. H. E. Haralambides, The economics of bulk shipping pools. *Marit. Policy Manag.* **23**, 221–237 (1996).

50. J. H. Ford, C. Wold, D. Currie, C. Wilcox, Incentivising change to beneficial ownership and open registers—Holding flag states responsible for their fleets and costs of illegal fishing. *Fish Fish.* **23**, 1240–1248 (2022).

51. G. Carmine, J. Mayorga, N. A. Miller, J. Park, P. N. Halpin, G. O. Crespo, H. Österblom, E. Sala, J. Jacquet, Who is the high seas fishing industry? *One Earth* **3**, 730–738 (2020).

52. H. Österblom, J.-B. Jouffray, C. Folke, B. Crona, M. Troell, A. Merrie, J. Rockström, Transnational corporations as 'keystone actors' in marine ecosystems. *PLOS ONE* **10**, e0127533 (2015).

53. J. Virdin, T. Vegh, J.-B. Jouffray, R. Blasiak, S. Mason, H. Österblom, D. Vermeer, H. Wachtmeister, N. Werner, The Ocean 100: Transnational corporations in the ocean economy. *Sci. Adv.* **7**, eabc8041 (2021).

54. E. Havice, L. M. Campbell, L. Campling, M. D. Smith, Making sense of firms for ocean governance. *One Earth* **4**, 602–604 (2021).

55. G. Whittaker, Going in for the krill, *TradeWinds* (2014); www.tradewindsnews.com/twplus/going-in-for-the-krill/1-1-338127.

56. Greenpeace International, Fishy Business: how transshipment at sea facilitates illegal, *unreported and unregulated fishing that devastates our oceans* (2020).

57. H. Thanopoulou, Bulk reefer market economics in a product life cycle perspective. *Marit. Policy Manag.* **39**, 281–296 (2012).

58. K. L. Seto, N. A. Miller, D. Kroodsma, Q. Hanich, M. Miyahara, R. Saito, K. Boerder, M. Tsuda, Y. Oozeki, O. Urrutia, Fishing through the cracks: The unregulated nature of global squid fisheries. *Sci. Adv.* **9**, eadd8125 (2023).

59. C. van der Geest, Transshipment: Strengthening tuna RFMO transshipment regulations (Tech. Rep. 2023-06, International Seafood Sustainability Foundation, 2023).

60. Ministerio De Desarrollo Agropecauario, Technical Circular/ARAP/IFA/TC/012/2020 (Ministerio De Desarrollo Agropecauario, 2020).

61. European Union (EU), Commission Implementing Regulation (EU) 2019/626 of 5 March 2019 Concerning Lists of Third Countries or Regions Thereof Authorised for the Entry into the European Union of Certain Animals and Goods Intended for Human Consumption, Amending Implementing Regulation (EU) 2016/759 as Regards These Lists (Text with EEA Relevance) (EU, 2019); http://data.europa.eu/eli/reg_impl/2019/626/oj/eng.

62. MRAG Asia Pacific, ICCAT Transhipment Business Ecosystem Study (MRAG Asia Pacific, 2020); https://mragasiapacific.com.au/projects/iccat-transhipment-business-ecosystem-study/.

63. European Commission, Non-EU countries authorised establishments - European Commission (European Commission, 2016); https://food.ec.europa.eu/safety/biological-safety/food-hygiene/non-eu-countries-authorised-establishments_en.

64. B. M. Pettersen, Russisk lasteskip ble nektet adgang–her får de norsk hjelp (NRK, 2024); www.nrk.no/nordland/russisk-lasteskip-ble-nektet-adgang-i-frykt-for-spionasje-_-her-far-de-norsk-hjelp-1.16902819.

65. Association of Reponsible Krill Harvesting Companies (ARK), ARK Voluntary Measures (ARK, 2022); www.ark-krill.org/ark-voluntary-measures.

66. S. Orofino, G. McDonald, J. Mayorga, C. Costello, D. Bradley, Opportunities and challenges for improving fisheries management through greater transparency in vessel tracking. *ICES J. Mar. Sci.* **80**, fsad008 (2023).

67. W. Swartz, A. M. Cisneros-Montemayor, G. G. Singh, P. Boutet, Y. Ota, AIS-based profiling of fishing vessels falls short as a "proof of concept" for identifying forced labor at sea. *Proc. Natl. Acad. Sci.* **118**, e2100341118 (2021).

68. Organisation for Economic Cooperation and Development (OECD), OECD Review of Fisheries 2020 (OECD, 2020); www.oecd-ilibrary.org/agriculture-and-food/oecd-review-of-fisheries-2020_7946bc8a-en).

69. IMO, *Resolution MSC.160(78) - Adoption of the IMO Unique Company and Registered Owner Identification Number Scheme - (Adopted on 20 May 2004)*" (2004); www.imorules.com/MSCRES_160.78.html.

70. J.-B. Jouffray, B. Crona, E. Wassénius, J. Bebbington, B. Scholtens, Leverage points in the financial sector for seafood sustainability. *Sci. Adv.* **5**, eaax3324 (2019).

71. OECD, Ownership and control of ships (Maritime Transport Committee, 2003).

72. G. Vuillemey, Evading corporate responsibilities: Evidence from the shipping industry (2020); https://ssrn.com/abstract=3691188.

73. E. Plomaritou, A. Papadopoulos, *Shipbroking and Chartering Practice*, (Routledge, 2017).

74. GREENSEA, Why GreenSea (2023); www.greensea.be/why-greensea.

75. MRAG, WCPO Transhipment Business Ecosystem Study (2019); https://mragasiapacific.com.au/projects/iccat-transhipment-business-ecosystem-study/.

76. AIS-based profiling of fishing vessels falls short as a "proof of concept" for identifying forced labor at sea" (1984); www.frigoship.de/index.html.

77. H. Österblom, C. Folke, Globalization, marine regime shifts and the Soviet Union. *Philos. Trans. R. Soc. Lond. B* **370**, 278 (2015).

78. L. G. Soldéus. *Fiskköpare, sumpskeppare och deras seglande sumpar: Handeln med levande fisk i Stockholm och i skärgårdarna* (Soldeko, 2013).

79. G. Sundnes, On the transport of live cod and Coalfish. *ICES J. Mar. Sci.* **22**, 191–196 (1957).

80. Nærings- og fiskeridepartementet, Forskrift Om Landings- og sluttseddel (landingsforskriften) (2015); https://lovdata.no/dokument/SF/forskrift/2014-05-06-607.

81. Nærings- og fiskeridepartementet, Forskrift om gjennomføring av fiske, fangst og høsting av viltlevende marine ressurser (høstingsforskriften) (2022); https://lovdata.no/dokument/SF/forskrift/2021-12-23-3910/KAPITTEL_7#%C2%A738.

82. Nærings- og fiskeridepartementet, Forskrift om elektronisk rapportering for norske fiske- og fangstfartøy under 15 meter (forskrift om kystfiskeappen) (2021); https://lovdata.no/dokument/SF/forskrift/2014-12-19-1822/%C2%A75#%C2%A75.
